# microRNAs Are Key Regulators in Chronic Lung Disease: Exploring the Vital Link between Disease Progression and Lung Cancer

**DOI:** 10.3390/jcm8111986

**Published:** 2019-11-15

**Authors:** Mathew Suji Eapen, Kielan Darcy McAlinden, Stephen Myers, Wenying Lu, Sukhwinder Singh Sohal

**Affiliations:** Respiratory Translational Research Group, Department of Laboratory Medicine, School of Health Sciences, University of Tasmania, Launceston, Tasmania 7248, Australia; mathew.eapen@utas.edu.au (M.S.E.); kielan.mcalinden@utas.edu.au (K.D.M.); stephen.myers@utas.edu.au (S.M.); Wenying.Lu@utas.edu.au (W.L.)

**Keywords:** microRNA, COPD, lung cancer

## Abstract

microRNAs (miRNAs) bind to mRNAs and inhibit their expression through post-transcriptionally regulating gene expression. Here, we elaborate upon the concise summary of the role of miRNAs in carcinogenesis with specific attention to precursor respiratory pathogenesis caused by cigarette smoke modulation of these miRNAs. We review how miRNAs are implicated in cigarette-smoke-driven mechanisms, such as epithelial to mesenchymal transition, autophagy modulation, and lung ageing, which are important in the development of chronic obstructive pulmonary disease and potential progression to lung cancer. Extracellular vesicles are key to inter-cellular communication and sharing of miRNAs. A deeper understanding of the role of miRNAs in chronic respiratory disease and their use as clinical biomarkers has great potential. Therapeutic targeting of miRNAs may significantly benefit the prevention of cancer progression.

The recent review article from Fujii et al. provides a concise and comprehensive summary of research uncovering the role of microRNAs (miRNAs) in carcinogenesis with specific attention on cigarette smoke modulation of these miRNAs [1]. In short, miRNAs bind to mRNAs and inhibit their expression through post-transcriptionally regulating gene expression [2]. Cigarette smoke regulates the expression of various miRNAs (e.g., epigenetic repression of miR-487b, reduced expression of mir-218), promoting smoking-induced carcinogenesis either through the silencing of anticancer molecules or upregulation of genes involved in lung cancer [3,4].

We are particularly interested in this process involving cigarette-smoke-driven lung carcinogenesis and applaud the writers of this review on their excellent summary of the findings on miRNAs and the development of lung cancer. Of note, tobacco cigarette smoking is the primary cause of chronic obstructive pulmonary disease (COPD) and is expected to be the third leading cause of global mortality by 2030, with COPD closely associated with the development of lung cancer [5]. The risk of developing lung cancer is far more significant for those suffering from COPD who smoke than those without [6], and along with underlying pathophysiological changes, COPD is most likely to be the strongest driver of lung cancer [5]. In the United States, approximately 80% of lung-cancer-related deaths were linked to smoking in 2012 [7]. Repeated exposure to cigarette smoke can also result in chronic stress from free radicals [8,9] and cellular reprogramming events such as epithelial to mesenchymal transition (EMT), which is highly active in both COPD and lung cancer patients [10,11,12,13,14,15,16,17]. Fujii et al. showed the promotion of EMT via miR-331-3p, contributing to the progression of prostate cancer [18]. miR-331-3p is over-expressed in asbestos-related lung cancer samples [19]. miR-200 is associated with tumour suppression through the binding to zinc finger E-box-binding homeobox 1 (ZEB1) and E-cadherin transcriptional repressor, resulting in the inhibition of EMT [20]. Lung cancer cell metastasis has been shown to be regulated by miR-200 expression [21]. Activation of the autophagy proteins and overall elevation of autophagy upon cigarette smoke stimulation carries significance in COPD pathogenesis and the potential progression to lung cancer [22,23]. miR-155 has been shown to be involved in the inhibition of autophagy, boosting the immune system, recruitment of tumour-infiltrating lymphocytes, and improving chemosensitivity [24]. We are witnessing a gradual decline in global cigarette smoking prevalence [25]. However, the rise in electronic nicotine delivery systems (ENDS) is concerning [26]. Exposure to electronic cigarette vapour has been shown to induce systemic inflammation and multi-organ fibrosis in mice [27], with short-term exposure linked to an increase in angiogenesis [28]. We highlight that electronic cigarette exposure also affects the lungs and may have a potential carcinogenic nature [29,30]. Nitrosamines were recently identified in electronic cigarette vapour, which implicates that inhalation could potentiate in lung cancer via induced DNA damage [31]. 

Fujii et al. further illustrates the potential role of smoking-related miRNAs in ageing and their susceptibility to lung and other forms of cancer [1]. Cellular ageing is known to cause tissue metabolic homeostasis imbalances, tilting toward a more catabolic mechanism, which leads to progressive declines in structure and function, until their final demise [32]. Several factors define the progression of ageing including increased genomic instability/mutation, increase in telomerase activity vis-à-vis a decrease in telomere size, epigenetics, dysregulation of autophagy, increase urea cycle activity, increased cellular senescence and associated inflammation, apoptosis, and functional alteration in cell communication [33]. The genome-wide assessment has confirmed that decrease in miRNAs occurs during ageing and these changes, could increase the risk in the tumorigenic activity. For example, downregulation of cancer-associated miRNAs, such as miR-103, -107, -128, and -221, was observed in an aged compared to a younger population, and repression of these miRNA was associated with increased cancer risk [34]. In the younger smoking population and COPD patients, lung ageing could play a crucial role in the overall dynamics associated with disease progression and hence dysregulation in miRNA is likely to be symptomatic in these patient populations. 

Oxidants and toxins present in tobacco smoking induce considerable DNA damage, which increases the DNA damage response (DDR), causing pathophysiology ageing disorders such as cardiovascular disease (CVD) and cancer, which are characteristic comorbidities often diagnosed within the COPD patient population [5]. Suppression of miRNA-126 expression is known to play an essential role in the development of CVD and several types of cancer, including lung cancer. miRNA126 expression in epithelial and endothelial cells from smokers and COPD patients were reduced, which was also associated with increased DDR, cellular senescence, and lung tissue dysfunction [35]. A more recent genome-wide study in the whole blood of COPD patients identified two blood-born microRNAs (miR-150-5p and miR-320b) that could efficiently predict patient survival. Higher expression of miR-150-5p showed increase survivability in COPD patients compared with patients with the lowest expression of the miRNA [36]. 

Inter-cell communication occurs via the transfer of extracellular vesicles (EVs) [37]. Based on the size and origin, EVs are distinguished into exosomes, ectosomes, and apoptotic bodies. In physiological conditions, they play a crucial role in maintaining tissue homeostasis. Exosomes are typically generated by inward budding of the membrane (endocytosis), subsequently form multivesicular bodies, and are released by exocytosis [38]. Ectosomes are formed by outward blebbing from the plasma membrane and are released by proteolytic cleavage from the cell surface. Both exosomes and ectosomes play differential roles [38]: exosomes carry vital cellular information in the form of embedded functional proteins, RNA, and DNA; ectosomes carry cell surface receptors and ligands that could externally activate cells by effectively inducing cellular signalling mechanisms.

EVs are also known to contain an array of miRNAs that are internalised or transferred into neighbouring cells and can produce similar phenotypic effects as parent cells [37]. Such EV-based cellular transformation has been demonstrated in an *invitro* co-culture study using human bronchial epithelial cells (HBEC) and a lung fibroblast model of COPD. Cigarette smoke extract induced HBEC-cells-derived exosomes carrying miRNA-210 was found to promote the transformation of the lung fibroblast into its more active and aggressive form, myofibroblast [39]. miRNA210 significantly decreased autophagy induction in COPD patients by silencing ATG7, an autophagy regulator. Inhibition of ATG7 was also observed to transform lung fibroblast to myofibroblast [39]. The abnormal increase in myofibroblast number has been widely implicated in pathological lung tissue remodelling via the accumulation of extracellular matrix proteins such as collagen and fibronectin [40]. 

Fujii et al. further explored the specific role of miRNA expression in driving various mechanisms associated with cancer developments [41]. miRNAs are now known to influence cancer in two ways: by regulating the expression of protein-coding oncogenes and tumour suppressors, such as ALK, TP53, VEGF family, E-cadherin; or by acting as oncogenes and tumour suppressors, such as the let-7, miR-21, and miR34 family [42]. For example, miR-126 is a cancer-suppressive miRNA that can decrease the expression of vascular endothelial growth factor-A (VEGF-A). VEGF up-regulation is observed in many carcinomas including lung carcinoma, and this primarily occursvia the suppression of miR-126 expression [43]. Cigarette smoke exposure is a major factor that affects the expression of miRNAs, especially during the early stage. Tumour suppressor miRNAs are mainly downregulated due to cigarette smoke exposure [41]. Smoking could also induce single nucleotide polymorphism (SNP) mutations in miRNA genes, which lead to their downregulation [44]. However, one study showed that two miRNAs, mir-138 and let-7c, were significantly downregulated in non-smokers with lung cancer. Another study similarly reported that reduced let-7 expression occurred early during lung carcinogenesis [45]. miRNAs have been proven to be most efficient in assisting with identifying tumour histology and predicting patient outcomes [46]. For instance, studies reported that miRNA-486-5p is downregulated and correlated with ankyrin1 (ANK1) expression in non-small cell lung cancer (NSCLC) [47]. The study also found that ANK1 methylation is more prevalent in adenocarcinoma compared with squamous cell carcinoma.

Reduced miR-486-5p expression actively contributes to lung cancer progression. Thus, it could be potentially used as a prognostic biomarker that could be reliably detected in easily accessible samples such as serum and sputum [48,49,50]. miRNAs have also been shown to play a crucial role in lung cancer chemoresistance by affecting autophagic flux and expression of both programmed cell death-1 (PD-1) and ligand PD-L1 (PD-L1) through post-transcriptional regulation [51,52,53]. PD-1 plays a crucial role in chemoresistance by suppressing the immune response during lung tumorigenesis [54,55]. Specifically, PD-1, as a cell surface receptor present on T cells and pro-B cells, binds its ligand PD-L1 and PD-L2 and triggers a net immunosuppressive effect that allows tumour cells to evade immune detection and destruction [56]. Accumulating evidence suggests that miRNAs can modulate several molecular mechanisms involved in tumour progression, autophagy activation, and metastasis. The clinical benefit of these mechanisms will be using these miRNA and other prognostic miRNA signatures as disease fingerprints and implementing them as potential early detection biomarkers, complementing concurrent lung cancer screening [51,52,53]. 

As our understanding grows in the area of cigarette-smoke-induced disease carcinogenesis, several other potentially deadly lifestyles have emerged, which could further contribute to disease vulnerability in the human population. We think a deeper understanding of the role of miRNAs in chronic lung pathogenesis will prove ideal in the fight against cancer progression.
